# Primary myogenesis in the sand lizard (*Lacerta agilis*) limb bud

**DOI:** 10.1007/s00427-019-00635-7

**Published:** 2019-06-18

**Authors:** Damian Lewandowski, Magda Dubińska-Magiera, Arnold Garbiec, Małgorzata Daczewska

**Affiliations:** Institute of Experimental Biology, Department of Animal Developmental Biology, University of Wroclaw, Sienkiewicza 21, 50-335, Wroclaw, Poland

**Keywords:** Limb bud, Myogenesis, Reptiles, MRFs, Pax3/Pax7, Lbx

## Abstract

Our studies conducted on reptilian limb muscle development revealed, for the first time, early forelimb muscle differentiation at the morphological and molecular level. Sand lizard skeletal muscle differentiation in the early forelimb bud was investigated by light, confocal, and transmission electron microscopy as well as western blot. The early forelimb bud, filled with mesenchymal cells, is surrounded by monolayer epithelium cells. The immunocytochemical analysis revealed the presence of Pax3- and Lbx-positive cells in the vicinity of the ventro-lateral lip (VLL) of the dermomyotome, suggesting that VLL is the source of limb muscle progenitor cells. Furthermore, Pax3- and Lbx-positive cells were observed in the dorsal and ventral myogenic pools of the forelimb bud. Skeletal muscle development in the early limb bud is asynchronous, which is manifested by the presence of myogenic cells in different stages of differentiation: multinucleated myotubes with well-developed contractile apparatus, myoblasts, and mitotically active premyoblasts. The western blot analysis revealed the presence of MyoD and Myf5 proteins in all investigated developmental stages. The MyoD western blot analysis showed two bands corresponding to monomeric (mMyoD) and dimeric (dMyoD) fractions. Two separate bands were also detected in the case of Myf5. The observed bands were related to non-phosphorylated (Myf5) and phosphorylated (pMyf5) fractions of Myf5. Our investigations on sand lizard forelimb myogenesis showed that the pattern of muscle differentiation in the early forelimb bud shares many features with rodents and chicks.

**Electronic supplementary material:**

The online version of this article (10.1007/s00427-019-00635-7) contains supplementary material, which is available to authorized users.

## Introduction

Vertebrate limb muscle differentiation and growth are one of the best known developmental processes, and this knowledge is obtained from chick and mouse embryonic studies. The appearance of buds along the lateral body wall is the first step of limb development (Martin 1998). Both forelimbs and hind limbs develop from the lateral mesodermal plate and grow into three main axes controlled by different signaling centers. The differentiation along the proximal–distal axis (from girdles to digits) is controlled by the apical ectodermal ridge (AER). The primary function of the AER is to maintain the distal cells in the mesenchymal core of the limb bud mitotically active and undifferentiated, both which are necessary for further limb growth (Kengaku et al. 1998; Kawakami et al. 2001). The posteriorly placed zone of polarizing activity (ZPA) is the site of sonic hedgehog (SHH) expression responsible for the anterior–posterior (from the 1st to the 5th digit) axis (Aono and Ide 1988). The third dorsal–ventral axis is regulated by wingless 7a (WNT7a) signaling from the ectoderm (Church and Francis-West 2002; Zeller et al. 2009; Butterfield et al. 2010).

The limb buds are composed of undifferentiated mesenchymal cells covered by an ectodermal cell layer. Mesenchymal cells of limb buds express one of the T-box transcription factor (TBX) family genes: TBX5 or TBX4 in the forelimb and hind limb respectively (Gibson-Brown et al. 1996). The mentioned transcription factors upregulate fibroblast growth factor 10 (*Fgf10*) expression in mesenchymal cells. FGF10 induces *Fgf8* expression in the overlying ectoderm (Ohuchi et al. 1997; Xu et al. 1998; Sekine et al. 1999; Ng et al. 2002; Agarwal et al. 2003; Minguillon et al. 2012; Nishimoto et al. 2015). The interactions between the mesenchyme and the ectoderm play a crucial role in the maintenance of mesenchymal cells’ proliferative activity and limb growth (Dealy et al. 1997)

In tetrapods, three parts of the limb can be distinguished: the stylopodium (arm/thigh), the zeugopodium (forearm/crus), and the autopodium (hand/foot). All three regions are present in quadrupedal tetrapods, with a number of individual bone elements varying between species (Petit et al. 2017).

In vertebrates, somites are the source of skeletal muscle precursors. During embryogenesis, somites differentiate into the dermomyotome, myotome, and sclerotome. Subsequently, the dermomyotome can be divided into epaxial and hypaxial parts with the ventro-lateral (VLL) and dorso-medial (DML) lips. The epaxial dermomyotome gives rise to the back musculature, whereas the limb skeletal muscles are formed by the cells derived from the hypaxial part of the dermomyotome. It has been shown that in mouse muscle progenitor cells express paired homeobox transcription factor 3 (Pax3) and paired homeobox transcription factor 7 (Pax7) proteins (Relaix et al. 2005). The mesenchymal cells located in limb buds differentiate into numerous tissues (including skeletal muscle tissue). The VLL is the source of limb muscle progenitor cells. The process of limb muscle differentiation shares similarities with trunk muscle development (premyoblasts divide mitotically, myoblasts withdraw from cell cycle and fuse to form multinucleated myotubes which differentiate into mature muscle fibers, MRF regulatory control) (reviewed by Bentzinger et al. 2012). The VLL cells undergo epithelial–mesenchymal transition, which leads to their migration into the limb bud. Simultaneously, migrating progenitor cells are characterized by proliferative activity, which results in an increase of the cell population and allows differentiation into striated muscles to begin (Christ and Ordahl 1995; Gros and Tabin 2014; Christ and Brand-Saberi 2002).

Hypaxial cell deepithelialization and migration are genetically regulated by numerous proteins, e.g., Pax3 (the marker of progenitor muscle cells detected in the dermomyotome and migrating cells) or tyrosine–protein kinase Met/c-Met (detected in progenitor muscle cells deepithelialized from the hypaxial dermomyotome) (Bober et al. 1994; Goulding et al. 1994; Williams and Ordhal 1994; Daston et al. 1996; Bladt et al. 1995). It is well known that the presence of ladybird homeobox transcription factor 1 (Lbx1) in migrating cells is unequivocal evidence of the migration of progenitor muscle cells (Jagla et al. 1995; Gross et al. 2000). In chicken and mammals, two *Lbx* genes were found, whereas zebrafish has three *Lbx* genes. In contrast, *Xenopus tropicalis* and lamprey have just one *Lbx* gene (Wotton et al. 2008, 2015). Recent studies revealed that the expression pattern of these genes in vertebrates is different during development. In mammals, *Lbx2* is expressed in the urogenital system, eye, and brain, whereas the two chicken and three zebrafish genes are co-expressed in migratory muscle precursors (Chen et al. 1999). It is hypothesized that *Lbx* genes in vertebrates are the result of the genome duplication during evolution. However, it is controversial if the genome duplication occurred before or after the gnathostome–agnathan divergence (reviewed by Wotton et al. 2015)

The muscle progenitor cells migrate into the dorsal and ventral parts of limb buds and form myogenic pools. As limb bud growth proceeds, the population of mesenchymal cells splits into three subpopulations (stylopodium, zeugopodium, autopodium) along the long axis of the future limb. The differentiation of limb muscles shows a proximal–distal gradient, which is correlated with limb growth (Christ and Brand-Saberi 2002).

The expression of the myogenic regulatory factor (MRF) family starts when the progenitor muscle cells are localized in limb buds. The superficial progenitor muscle cells express Myf5, whereas MyoD-positive cells localize in deeper layers of the limb bud (Patel et al. 2002). Limb bud myogenesis starts when the MyoD-positive cells fuse with each other, leading to primary muscle fiber formation. The primary muscle fibers are believed to be a scaffold for the secondary muscle fibers and every future muscle. Their main functions are the maintenance of the type, shape, and localization of mature muscles (Stockdale 1992, 1997; Christ and Brand-Saberi 2002).

Limb myogenesis in rodents and chicks has been investigated in detail at the morphological and molecular levels. Despite some molecular differences in MRF expression during limb muscle development between rat, mouse, and chick (in rat, MyoD occurs first, whereas, in mouse and chick, Myf5 is the first transcription factor observed in early limb bud), studies showed that the pattern of muscle differentiation in the limb is evolutionarily conserved (Christ and Brand-Saberi 2002; Duprez 2002; Francis-West et al. 2003; Murphy and Kardon 2011; Lee et al. 2013). So far, reptilian limb muscle development has not been studied at a detailed molecular level. The aim of our studies was to demonstrate reptilian early forelimb muscle differentiation at the morphological and molecular levels.

## Materials and methods

### Study animals

Gravid females of the sand lizard, *Lacerta agilis*, were caught in Poland in the vicinity of Wrocław at the beginning of June 2016. All of the specimens used in the experiments were captured according to the Polish regulations concerning the protection of wild species (Journal of Laws 1991, No. 114 Item 492; Journal of Laws 200, No. 66 Item 802; Journal of Laws 2004, No. 112 Item 1183; Journal of Laws 2015, No. 133 Item 266). The Department of Animal Developmental Biology of the University of Wrocław obtained approvals from the Local Ethics Commission in Wrocław (77/2013) and the Polish Ministry of Environment (Ref. No.: WPN.6401.51.2016.IW.1). The animals were kept in vivaria in an open area, in conditions similar to those in the wild (similar temperature, ventilation, humidity, differentiated bedding, hiding places) until the eggs were laid, and then they were released into their native area. The sand lizard eggs (*n* = 33) after oviposition were carefully collected and were placed inside plastic boxes filled with moistened perlite (at 100% humidity) with ventilation holes. Holes in the bottom and top of containers ensured air circulation. The eggs were prevented from desiccating by moistening perlite with water (twice a day) which ensured constant humidity in containers. The eggs were incubated at 30 °C, reflecting the seasonal ambient temperatures in the wild. The developmental stages of embryos were estimated using the developmental table published by Peter (1904).

The collected embryos were anesthetized with tricaine methanesulfonate (MS-222; 500 μg/g of body weight) (Conroy et al. 2009), before being decapitated and dissected for further analysis (Journal of Laws 2015, No. 133 Item 266).

### Light and transmission electron microscopy

For light and electron microscopic examination, the embryonic body wall with limb buds (including differentiated muscle tissue) was fixed in modified Karnovsky fixative (1% paraformaldehyde [PFA] and 1% glutaraldehyde, in a 0.1 M phosphate buffer pH 7.2) for 24 h at 4 °C. The material was repeatedly rinsed with the same buffer and was postfixed for 2 h in a 1:1 mixture of osmium tetroxide-potassium ferricyanide (OsO_4_-K_3_Fe(CN)_6_). Following rinsing in the phosphate buffer, the material was dehydrated, first in a graded alcohol series and then in acetone, and was then embedded in epoxy resin Epon 812 (Sigma-Aldrich) (Luft 1961). The Epon blocks were cut on Leica Ultracut UCT (Leica, Wetzlar, Germany). Semithin sections (0.6 μm) were collected on glass slides and were stained with methylene blue in a 1% borax solution. The stained material was examined under an Olympus BX60 light microscope (Olympus). The ultrathin sections were collected on 200-mesh copper grids and were stained with uranyl acetate and lead citrate according to the standard protocol (Reynolds 1963), before being examined under the transmission electron microscope (TEM), Zeiss EM 900 (Carl Zeiss AG, Oberkochen, Germany; 80 kV).

### Immunofluorescence analysis

After the dissection and fixation of embryos (4% PFA in phosphate buffer saline, PBS for 45 min at room temperature), the samples were transferred to 30% sucrose in PBS for overnight incubation at 4 °C. Next, samples were embedded in optimal cutting temperature medium (OCT) and placed in a cryomold and frozen. The samples were cut into 10 μm sections in a cryostat (Leica) at − 24 °C and were placed on SuperFrost Plus slides and subjected to immunofluorescence staining.

Standard immunofluorescence reactions were carried out on tissue cryosections described in our previous paper (Lewandowski et al. 2017). The following primary antibodies were used: rabbit polyclonal anti-phospho-histone H3 (pSer10) (Sigma-Aldrich) at dilution of 1:200 in PBST, mouse monoclonal anti-Pax3 (Developmental Studies Hybridoma Bank) at dilution of 1:50 in PBST, mouse monoclonal anti-Pax7 (Developmental Studies Hybridoma Bank) at dilution of 1:50 in PBST, mouse monoclonal anti-Lbx2 (Abcam) at dilution of 1:200 in PBST. Additionally, the following secondary antibodies were used: goat anti-mouse IgG-FITC conjugated (Sigma-Aldrich) at dilution of 1:50 in PBST, goat anti-rabbit IgG TRITC conjugated (Sigma-Aldrich) at dilution of 1:50 in PBST, donkey anti-mouse IgG Cy5 conjugated (Jackson ImmunoResearch) at dilution of 1:100 in PBST, donkey anti-rabbit IgG Cy5 conjugated (Jackson ImmunoResearch) at dilution of 1:100 in PBST, donkey anti-rabbit IgG Cy3 conjugated (Jackson ImmunoResearch) at dilution of 1:100 in PBST. For the F-actin identification, Alexa Fluor 488–conjugated phalloidin and Alexa Fluor 546–conjugated phalloidin (Molecular Probes) were used at a dilution of 1:80 in PBS. The DNA was stained with 4,6-diamidino-2-phenylindole (DAPI; 0.2 μg/ml). For the imaging, an Olympus FluoView FV1000 confocal laser scanning microscope (Olympus) was used. The images were recorded by employing the Plan-Apochromat × 10, × 20, or × 40 objectives. Brightness and contrast adjustments were performed in the FV10-ASW_Viewer or in ImageJ.

### SDS-PAGE electrophoresis and western blot

The decapitated embryos and limb bud lysates were prepared, separated by SDS-PAGE, and analyzed by Western blot as described previously (Lewandowski et al. 2017). The membranes with separated and transferred proteins were incubated with the following primary antibodies: mouse monoclonal anti-Pax3 (Developmental Studies Hybridoma Bank) at dilution of 1:100, mouse monoclonal anti-Lbx2 (Abcam) at dilution of 1:200, rat monoclonal anti-α-actinin (Babraham Bioscience Technologies) at dilution of 1:250, mouse monoclonal anti-MyoD (Santa Cruz Biotechnology) at dilution of 1:200, rabbit polyclonal anti-Myf5 (GeneTex) at dilution of 1:200. Additionally, secondary antibodies were used: donkey anti-mouse IgG HRP conjugated (Jackson ImmunoResearch) at dilution of1:10000, donkey anti-rabbit IgG HRP conjugated (Jackson ImmunoResearch) at dilution of 1:10000, donkey anti-rat HRP conjugated (Jackson ImmunoResearch) at dilution of 1:10000. Membranes were then detected and documented with a chemiluminescent method using the Bio-Rad imaging system. The protein content in the Pax3, Lbx, MyoD, and Myf5 bands was then normalized according to the α-actinin content in each lane.

### LC-MS analysis

Homogenized samples were prepared as described by Mroczek et al. (2017). The limb bud lysates were separated by SDS-PAGE and stained by modified silver staining (Shevchenko et al. 1996). A stained portion of the gel (size of cutout bands corresponded to the size of bands detected in western blot technique) was cut out. Samples of proteins were sent for the identification by an LC-MS method in the Mass Spectrometry Laboratory, IBB PAS and searched with MASCOT (Matrix Science) against homemade protein database containing 538 entries including known Lbx sequences, downloaded 18.04.2019 from UniProt.

### Densitometric analysis of MyoD and Myf5 fractions

The ratio of different fractions of the tested proteins (monomeric vs. dimeric in the case of MyoD and non-phosphorylated vs. phosphorylated in the case of Myf5) was determined via densitometric measurement of the signal generated by a western blot analysis. Imaging software (Image Lab 6.0; Bio-Rad) was used to compare the signal generated by the bands detected on the membranes.

Statistical analyses were carried out using MS Excel. Student’s *t* test was used for comparisons of the percentage amounts (*n* = 5 for MyoD; *n* = 3 for Myf5). The results are reported in the graph, and *p* < 0.05 is considered statistically significant.

## Results

The sand lizard limb bud myogenesis was investigated at stages 21–23 by the use of a light microscope, confocal microscope, TEM, and Western blot.

**Fig. 1:**
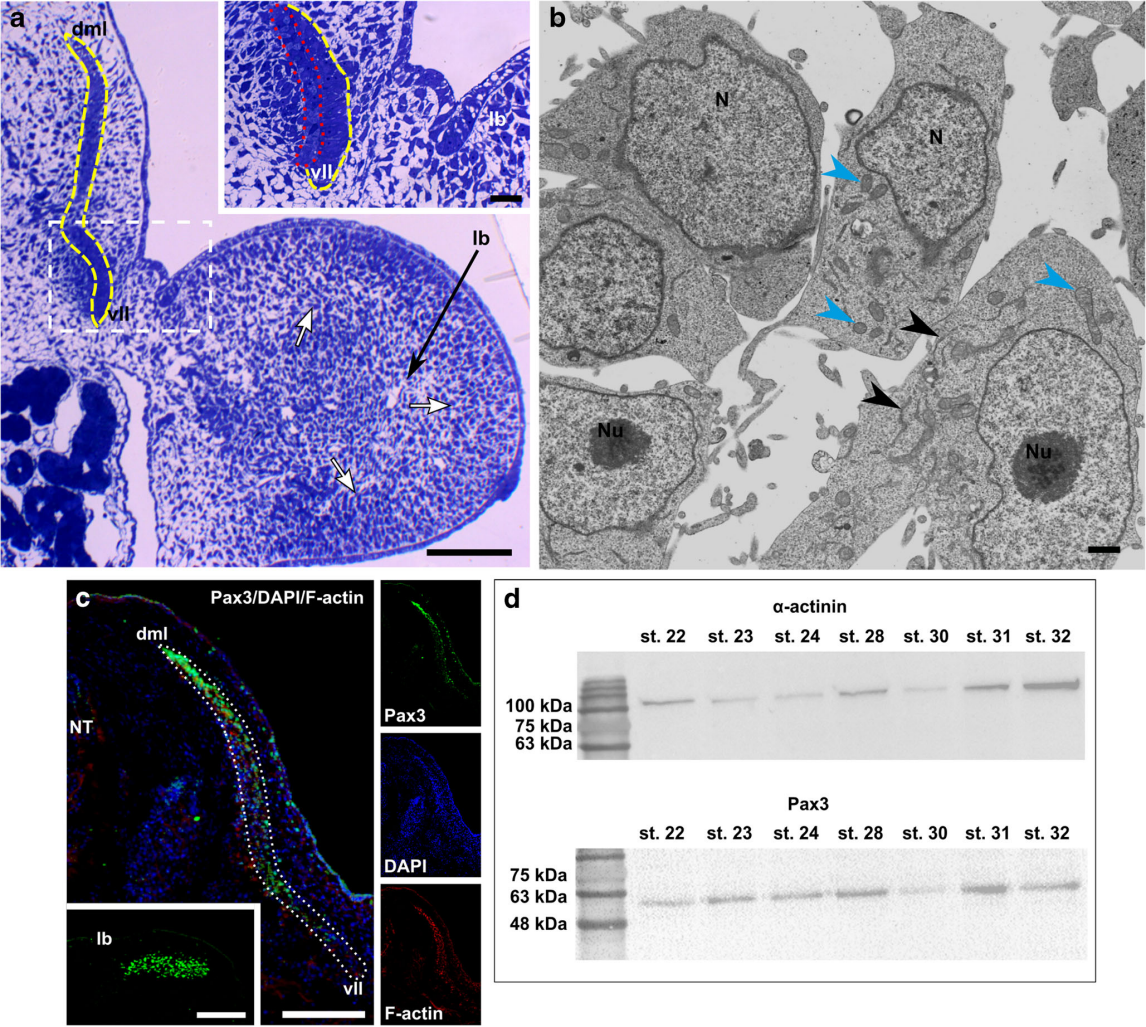
Limb bud muscle differentiation. **a** Stage 23. Cross section through embryo. Dermomyotome (yellow, dashed line), ventro-lateral (vll), and dorso-medial (dml) lips of dermomyotome. Note mesenchymal cells (white arrows) in developing limb bud (lb)*.* Transverse, semithin section, methylene blue staining, light microscope. Scale bar: 100 μm. Inset: Magnification of region marked by white, dashed frame. Myotome (red, dotted line), ventro-lateral (vll) lip of dermomyotome (yellow, dashed line), limb bud (lb). Transverse, semithin section, methylene blue staining, light microscope. Scale bar: 20 μm. **b** Stage 23. Ultrastructure of forelimb bud myogenic pool filled with mesenchymal cells. Nuclei (N), nucleoli (Nu), RER (black arrowheads), mitochondria (blue arrowheads). Transverse, ultrathin section. Scale bar: 1 μm. **c** Stage 23. Immunodetection of Pax3 protein (green) in dermomyotome (white, dotted line) and dorsal forelimb bud (lb). Nuclei (blue), F-actin (red), ventro-lateral (vll), and dorso-medial (dml) lips of dermomyotome, neural tube (NT). Transverse, cryosection, confocal microscope. Scale bar: 100 μm. Inset: Pax3-positive cells (green) in dorsal muscle mass of forelimb bud (lb). Transverse, cryosection, confocal microscope. Scale bar: 100 μm. **d** Western blot analysis of Pax3 protein expression in myotome during successive developmental stages (stages 22–32). Pax3 is marked together with the α-actinin band used as a loading control

During the early stage (stage 23) of sand lizard embryogenesis, the forelimb bud, surrounded by a monolayer of epithelial cells, is filled with mesenchymal cells (Fig. 1a). Mononucleated mesenchymal cells are irregularly shaped. Their cytoplasm, with a large nucleus, is rich in mitochondria and rough endoplasmic reticulum (RER) (Fig. 1b). The immunodetection of the Pax3 protein (a marker of muscle progenitor cells) revealed that this protein is present in the epithelial dermomyotome, in the VLL, DML, and in the myotome (Fig. 1c). At this stage of myogenesis, Pax3-positive muscle precursor cells are also observed in the dorsal muscle mass of the forelimb (Fig. 1c). The presence of Pax3 protein during trunk muscle myogenesis was confirmed by western blot analysis (stages 22–32) (Fig. 1d). No Pax7- (a marker of muscle progenitor and satellite cells) positive cells are detected in the *L. agilis* forelimb bud (stage 23), while in the *L. agilis* trunk Pax7-positive cells in the myotomes were previously observed at the same stage of embryogenesis (Rupik et al. 2012; Lewandowski, unpublished).

**Fig. 2:**
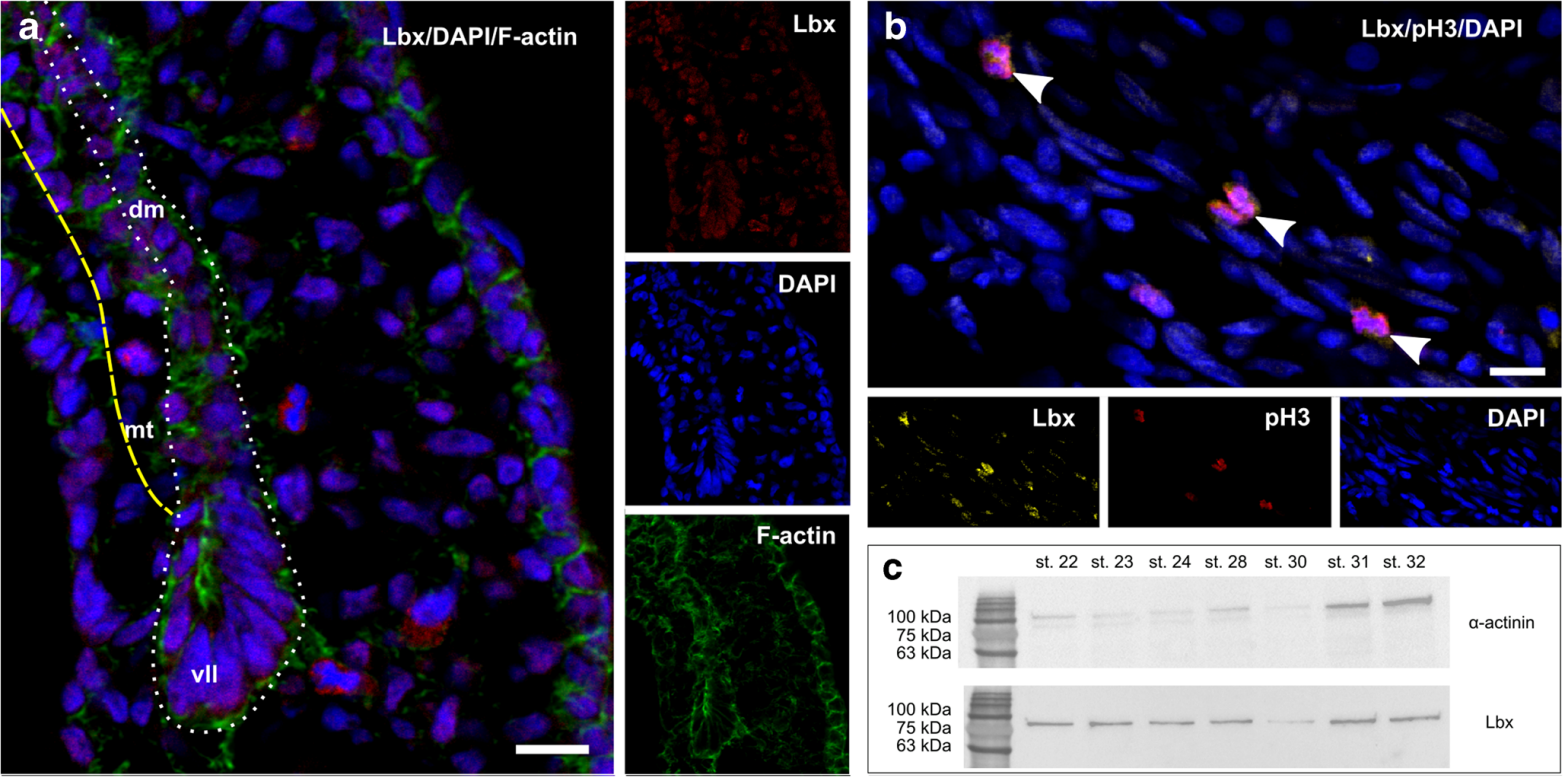
Expression of Lbx protein in myotome. **a** Stage 23. Immunodetection of Lbx-positive muscle progenitor cells (red) in the myotome (yellow, dashed line) and the vicinity of ventro-lateral lip (vll) of dermomyotome (white, dotted line). Nuclei (blue), F-actin (green). Transverse, cryosection, confocal microscope. Scale bar: 10 μm. **b** Stage 23. Immunodetection of mitotically active migrating muscle progenitor cells (white arrowheads) in the myotome. Lbx (yellow), phosphorylated histone H3 (red), nuclei (blue). Transverse, cryosection, confocal microscope. Scale bar: 10 μm. **c** Western blot analysis of Lbx protein expression during successive developmental stages (stages 22–32). Lbx is marked together with the α-actinin band used as a loading control

To demonstrate the presence of migratory muscle progenitor cells, immunodetection of Lbx protein was carried out in the trunk (myotome). In the myotome (stage 23), Lbx-positive cells were observed in the vicinity of the VLL (Fig. 2a). Lbx-positive cells were mitotically active, which was confirmed by the detection of phosphorylated histone H3 (Fig. 2b). Moreover, western blot analysis revealed that Lbx protein is present in trunk muscles in all investigated stages (stages 22–32) (Fig. 2c).

**Fig. 3:**
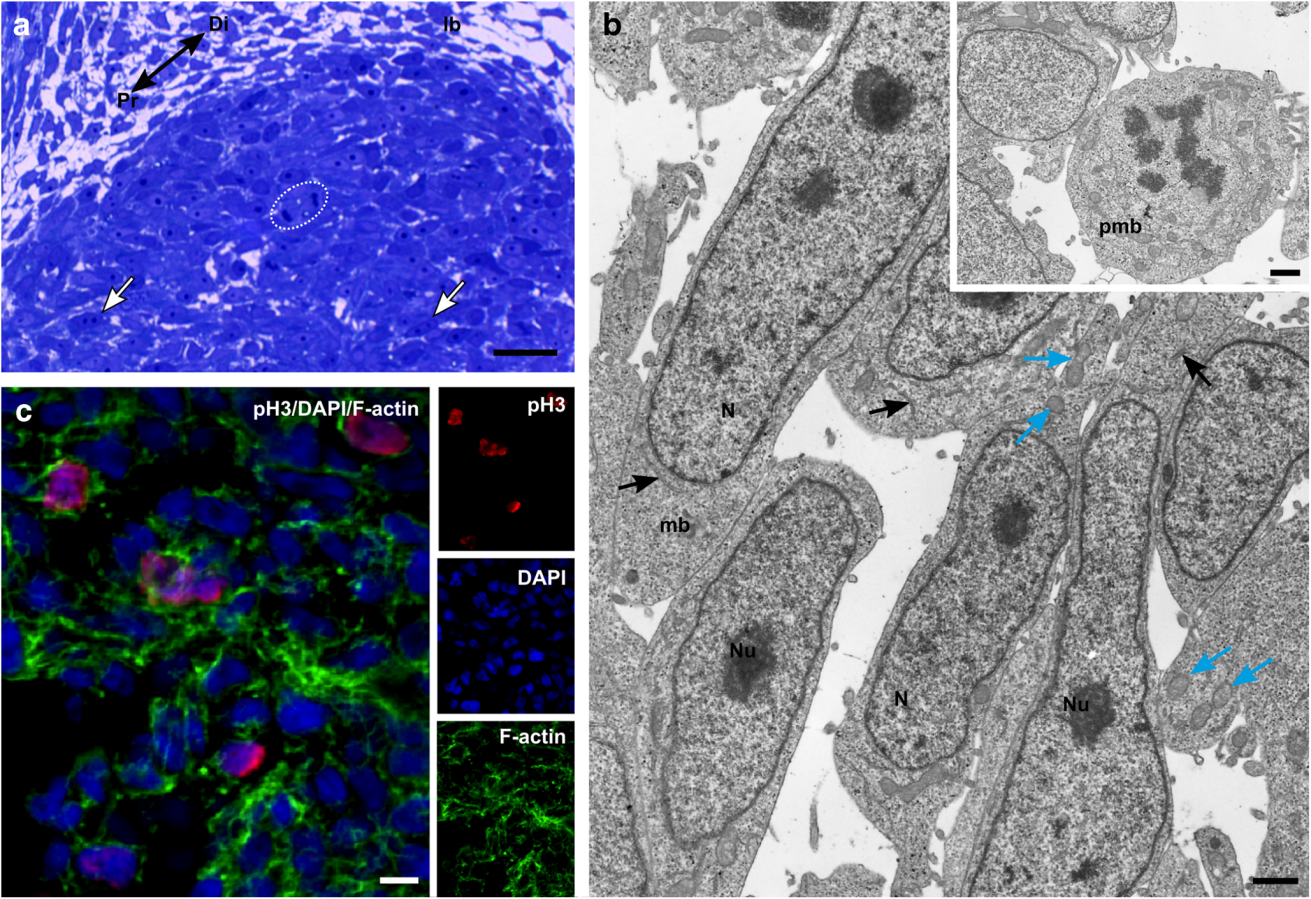
Myogenic pool in the forelimb. **a** Stage 24. Structure of forelimb bud (lb) with post-mitotic cells (white arrows) localized in proximal (Pr) part of the limb bud, mitotically active cells (circled) are in the distal (Di) part of the limb bud. Longitudinal, semithin section, methylene blue staining, light microscope. Scale bar: 20 μm. **b** Stage 24. Ultrastructure of forelimb bud. Elongated myoblasts (mb), nuclei (N), nucleoli (Nu), RER (black arrows), mitochondria (blue arrows). Longitudinal, ultrathin section. Scale bar: 1 μm. Inset: Ultrastructure of interphase (left) and mitotic (right) premyoblasts (pmb)*.* Longitudinal, ultrathin section. Scale bar: 1 μm. **c** Stage 24. Immunodetection of phosphorylated histone H3 (red). Nuclei (*blue*), F-actin (green). Transverse, cryosection, confocal microscope. Scale bar: 5 μm

At stage 24, in the forelimb, muscle mass mononucleated post-mitotic cells are observed in the proximal part, whereas mitotically active cells occupied a distal part of the developing forelimb (Fig. 3a). Their mitotic activity was confirmed by the detection of phosphorylated histone H3 (Fig. 3c). TEM analysis showed mitotically active premyoblasts and elongated post-mitotic myoblasts with centrally located large nuclei. The cytoplasm of myoblasts revealed the presence of numerous mitochondria and RER (Fig. 3b).

**Fig. 4:**
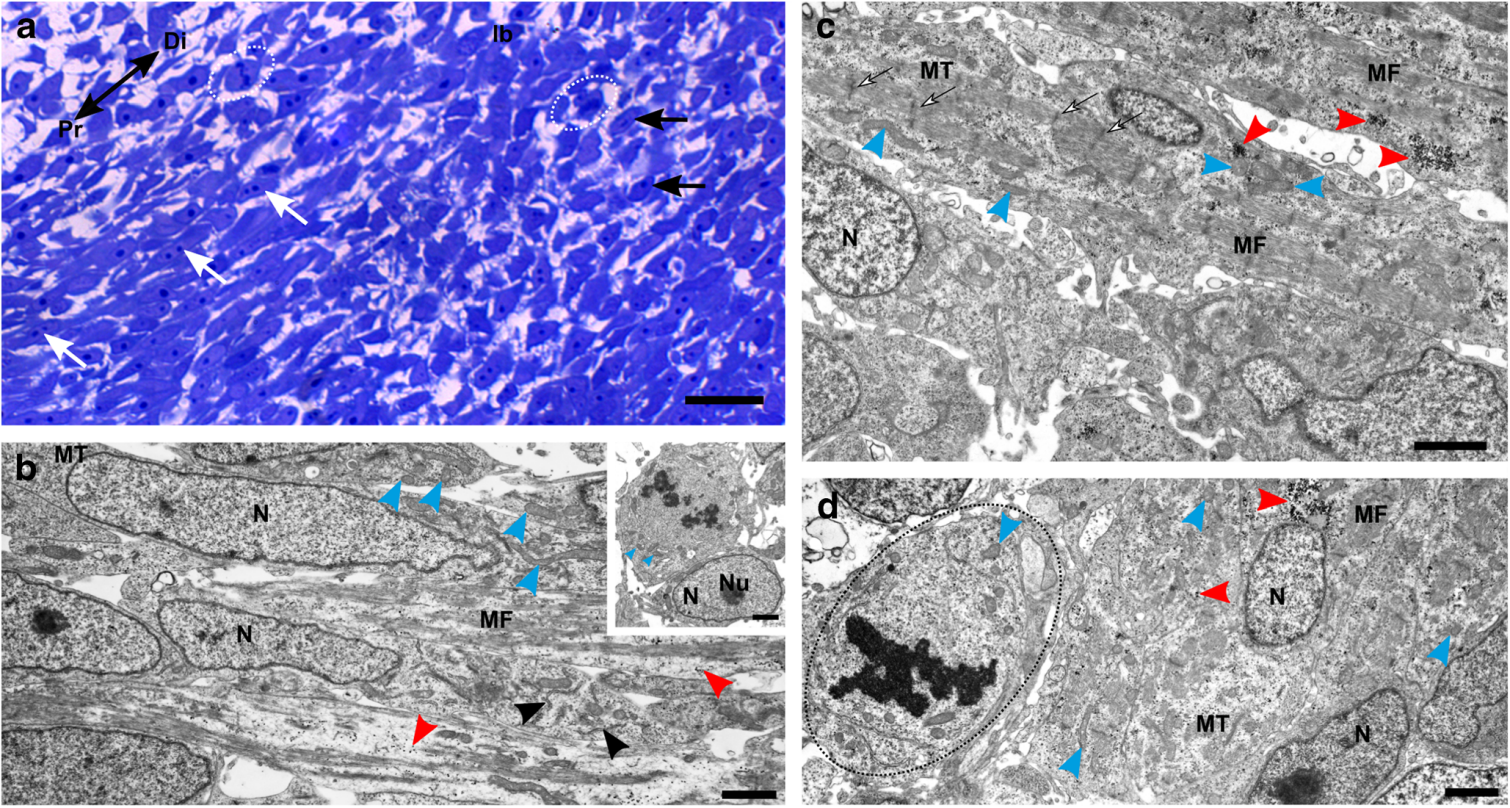
Structure of forelimb bud muscles. **a** Stage 28. Structure of forelimb bud (lb). Mononucleated cells (black arrows), elongated myoblasts (white arrows), premyoblasts (circled), proximal (Pr), distal (Di). Longitudinal, semithin section, methylene blue staining, light microscope. Scale bar: 20 μm. **b** Stage 28. Ultrastructure of forelimb bud myogenic pool. Nuclei (N), mitochondria (blue arrowheads), myotube (MT), myofibrils (MF), RER (black arrowheads), glycogen (red arrowheads). Longitudinal, ultrathin section. Scale bar: 1 μm. Inset: Ultrastructure of premyoblast. Nucleus (N), nucleolus (Nu), mitochondria (blue arrowheads). Longitudinal, ultrathin section. Scale bar: 1 μm. **c** Stage 32. Ultrastructure of myotube (MT) in the myogenic pool. Nucleus (N), mitochondria (blue arrowheads), myofibrils (MF), glycogen (red arrowheads), Z lines (white arrows). Longitudinal, ultrathin section. Scale bar: 2 μm. **d** Stage 32. Ultrastructure of forelimb myogenic pool filled with cells at different stages of differentiation: myotube (MT) and premyoblast (circled). Nucleus (N), mitochondria (blue arrowheads), myofibrils (MF), glycogen (red arrowheads). Longitudinal, ultrathin section. Scale bar: 2 μm

In the forelimb bud myogenic pool (stage 28), mononucleated myotubes are accompanied by premyoblasts. The characteristic feature of mononucleated myotubes at this developmental stage is irregularly arranged myofibrils. The myotube sarcoplasm contains numerous mitochondria, glycogen granules, and RER (Fig. 4a, b).

**Fig. 5:**
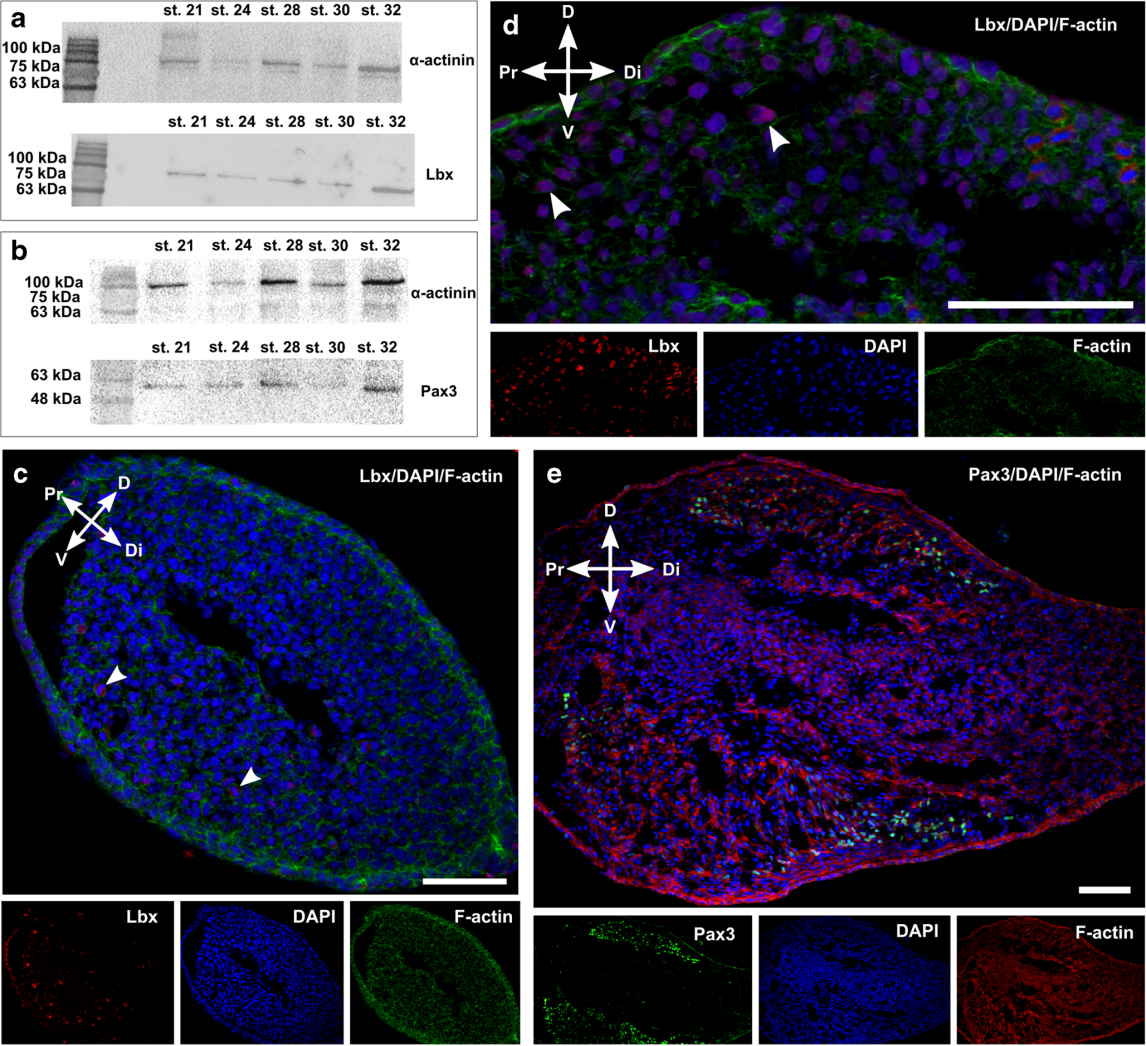
Expression of Lbx and Pax3 proteins in forelimb bud. **a**, **b** Western blot analysis of Lbx (**a**) and Pax3 (**b**) proteins expression during successive developmental stages (Stages 21–32). Lbx and Pax3 proteins are marked together with α-actinin bands used as a loading control. **c**, **d** Stage 30. Immunodetection of Lbx (white arrowheads and red) protein in ventral (**c**) and dorsal (**d**) part of forelimb bud. Nuclei (blue), F-actin (green), ventral (V), dorsal (D), proximal (Pr), distal (Di). Longitudinal, cryosection, confocal microscope. Scale bar: 50 μm. **e** Stage 30. Immunodetection of Pax3 (green) protein in ventral (V) and dorsal (D) part of forelimb bud. Nuclei (blue), F-actin (red), proximal (Pr), distal (Di). Longitudinal, cryosection, confocal microscope. Scale bar: 50 μm

Western blot analysis of the forelimb bud revealed the presence of Pax3 protein in all studied stages (stages 21–32) (Fig. 5a, b), which was confirmed by immunocytodetection of Lbx and Pax3 proteins in the dorsal and ventral limb bud (Fig. 5c–e).

To confirm the presence of Lbx protein in limb buds, peptide identification from silver-stained protein gel samples was performed. The comparison of obtained masses of peptides and their fragments with the NCBI protein sequences database was carried out using the MASCOT program. Obtained results revealed the presence of three peptides corresponding to *Xenopus laevis* Lbx1 (Fig. 1a, b; suppl.).

As limb bud muscle differentiation proceeded (stage 32), cells at different stages of differentiation can be observed: mitotic active premyoblasts, myoblasts, and multinucleated myotubes with well-developed contractile apparatus (Fig. 4d). The ultrastructure of myotube sarcoplasm shows that sarcomeres form characteristic repetitive units of light and dark bands with pronounced Z lines (Fig. 4c).

**Fig. 6:**
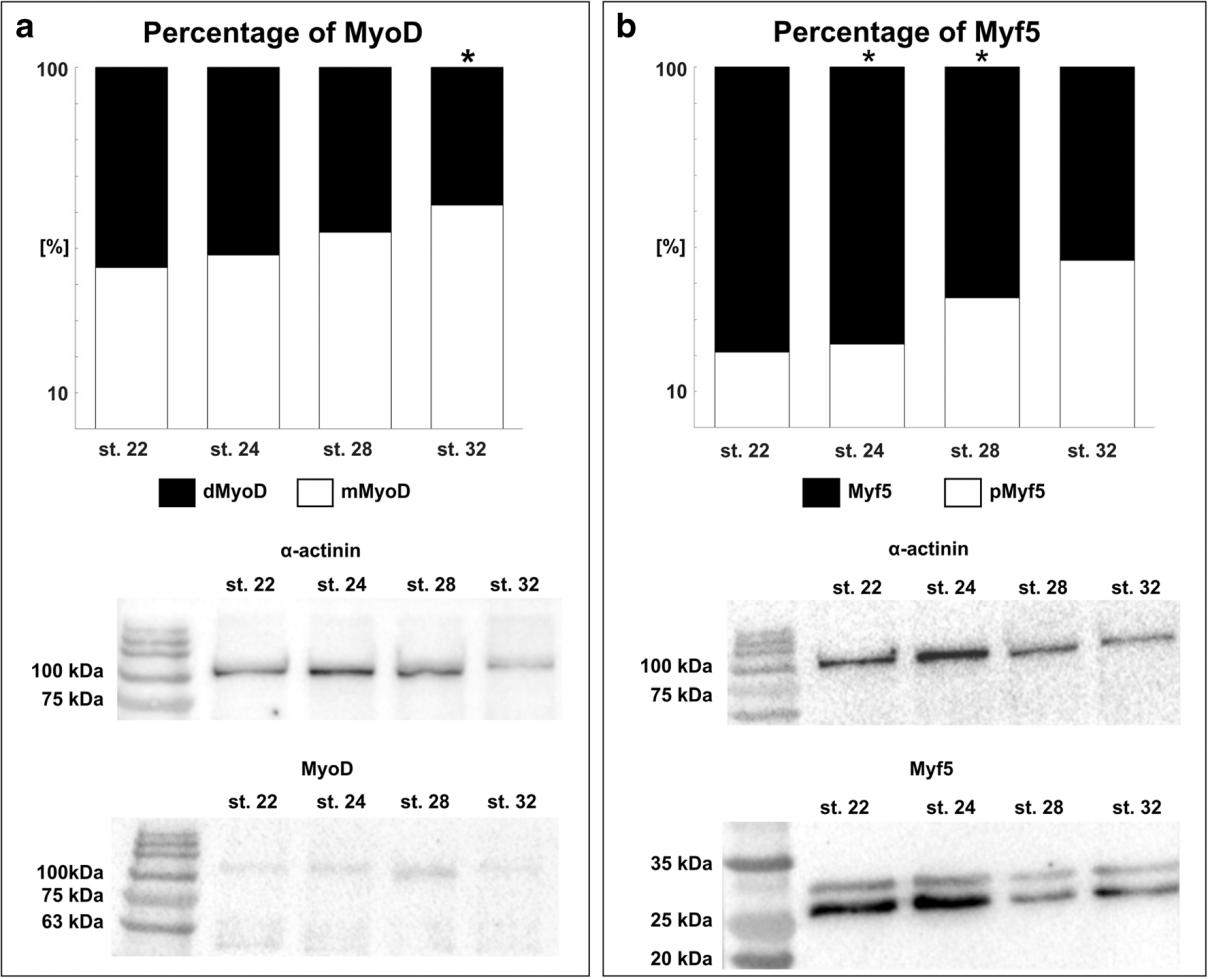
Expression of MyoD and Myf5 fractions during forelimb bud development. **a** Percentage amounts of different MyoD fractions (mMyoD, monomeric; dMyoD, dimeric) during successive developmental stages (stages 22–32). The analysis was based on densitometric measurement of Western blot detected bands. Statistically significant difference is indicated with an asterisk (*p* < 0.05; Student’s *t* test; *n* = 5). Western blot analysis of MyoD protein expression during successive developmental stages (stages 22–32). Note the presence of two bands corresponding to monomeric (MyoD) and dimeric (dMyoD) fraction of MyoD. MyoD is marked together with the α-actinin band used as a loading control. **b** Percentage amounts of different Myf5 fractions (Myf5, non-phosphorylated; pMyf5, phosphorylated) during successive developmental stages (stages 22–32). The analysis was based on densitometric measurement of Western blot detected bands. Statistically significant differences are indicated with asterisks (*p* < 0.05; Student’s *t* test; *n* = 3). Western blot analysis of Myf5 protein expression during successive developmental stages (stages 22–32). Note the presence of two bands corresponding to non-phosphorylated (Myf5) and phosphorylated (pMyf5) fractions of Myf5. Myf5 is marked together with α-actinin band used as a loading control

Our research revealed the presence of MyoD and Myf5 proteins in all investigated developmental stages (stages 22–32) (Fig. 6). The Western blot analysis of MyoD protein showed two bands corresponding to monomeric (mMyoD) and dimeric (dMyoD) fractions of MyoD (Fig. 6a). Since the intensity of detected bands was different in distinct developmental stages, we decided to estimate the ratio of monomeric and dimeric MyoD fractions in each investigated developmental stage. The conducted analysis showed statistically significant differences between the levels of mMyoD and dMyoD at stage 32 (*p* < 0.05) (Fig. 6a).

We also observed two separate bands during Myf5 Western blot analysis (Fig. 6b). The observed bands are related to non-phosphorylated (Myf5) and phosphorylated (pMyf5) fractions of Myf5 (Fig. 6b). Since the intensity of detected bands was different in distinct developmental stages, we decided to estimate the ratio of non-phosphorylated and phosphorylated Myf5 fractions. The conducted analysis showed statistically significant differences between Myf5 and pMyf5 at stages 24 and 28 (Fig. 6b).

## Discussion

### Structure of the early limb bud

Our investigations on sand lizard forelimb myogenesis showed that the pattern of muscle differentiation in the early forelimb bud shares many features with rodents and chicks. In sand lizard embryos, forelimb buds are surrounded by a monolayer of epithelial cells and present a core of mesenchymal cells. The myogenesis of the limb bud initially starts in the myogenic pool divided into the dorsal and ventral muscle mass. The process of muscle differentiation in the limb bud is asynchronous. In the myogenic pool, mitotically active progenitor muscle cells (premyoblasts), mononucleated myoblasts, mononucleated myotubes, and multinucleated muscle fibers are observed. As in rodents and chicks, sand lizard mononucleated myoblasts elongate and differentiate into mononucleated myotubes accompanied by mononucleated cells (premyoblasts or myoblasts). Lee et al. (2013) compared mononucleated myotubes observed in the early rodent and chick limb bud to mononucleated, so-called founder cells (FCs), found during *Drosophila melanogaster* somatic muscle development. In the fruit fly, FCs initiate myogenesis by fusion with non-specific cells, called fusion-competent myoblasts (FCMs). It is noteworthy that muscle morphology, localization in each hemisegment, innervation, and the connection site to the exoskeleton in *Drosophila* depend on the identity of FC genes (Baylies et al. 1998; Dobi et al. 2015). In contrast to vertebrate multi-fiber muscles, *Drosophila* muscles are mono-fiber. Our knowledge of mononucleated myotube gene identity in mammals and chicks is still very poor. It has been reported that the mouse adult limb muscles contain a different proportion of slow and fast fibers, but despite this they do not show notable differences in gene expression (Schafer and Braun 1999; Gross et al. 2000). Our ultrastructural studies on sand lizard muscles showed that not all myoblasts elongate and form multinucleated myotubes; some of them behave like FCMs observed in *Drosophila* myogenesis. These data show that muscle differentiation in a phylogenetically distant group of organisms may share unexpected similar features. To find some specific markers of mononucleated myotubes, detailed investigations at a molecular level should be carried out.

### Pattern expression of Pax3, Pax7, and Lbx proteins during early limb bud development

During chick and mammal limb myogenesis, Pax3 protein is essential for the survival of muscle progenitor cells and for their migration from VLLs into limb buds, whereas Pax7 is responsible for the specification of adult satellite cells at later developmental stages (Buckingham 2007). In the mentioned organisms, Pax3-positive progenitor cells were observed initially in the VLL of the dermomyotome and then in the dorsal and ventral muscle mass of the early forelimb bud (reviewed by Bentzinger et al. 2012; Zammitt 2017). Our investigation on sand lizard also showed Pax3-positive cells in the VLL and in the myogenic pool of the early forelimb bud. Sand lizard migrating cells also expressed Lbx protein. Based on studies carried out on mice, it was shown that Lbx1 and Pax3 are co-expressed in all migrating hypaxial muscle precursors. The authors assume that Lbx1 regulates their migration (Gross et al. 2000; Masselink et al. 2017). Previous studies demonstrated that in mice there exist two *Lbx* genes: *Lbx1* and *Lbx2*. *Lbx1* is expressed in hypaxial migratory progenitor cells, whereas *Lbx2* is observed in the central nervous and genitourinary systems (Chen et al. 1999). In *Danio rerio*, the presence of three *Lbx* genes were confirmed: *Lbx2*, *Lbx1a*, *Lbx1b*, *Lbx2* is expressed in fin buds and in the ventral part of the somite. In *Drosophila*, Lbx protein is engaged in the establishment of leg morphological and functional features (Maqbool et al. 2006). It was evidenced that in *D. rerio* Morpholino knockdown of *Lbx2* suppresses *MyoD* expression in fin buds. Of interest, the *Lbx2* gene is also implicated in myofibrillogenesis in both trunk and fin bud muscles (Ochi and Westerfield 2009). In our studies, to identify migratory muscle progenitors (MMPs), we used a commercial anti-Lbx2 antibody. LC-MS analysis showed the presence of three peptides corresponding to *Xenopus laevis* Lbx1. It is worthy to note that only *Lbx1* was found in veiled chameleon (*Chamaeleo calyptratus*) embryo transcriptome at the early limb bud stage (Pinto et al. 2019). Based on those results, our LC-MS analysis, and amino acids sequence comparison (epitope of anti-Lbx2 antibody vs. *Anolis carolinensis* vs. *Pogona vitticeps*) we assume that used anti-Lbx2 antibody recognizes the Lbx1 protein in *Lacerta agilis*. However, the presence of Lbx2 protein in *Lacerta agilis* cannot be excluded.

Our studies showed for the first time the presence of Lbx protein during sand lizard limb bud myogenesis. We strongly believe that the mentioned protein is engaged in the acquisition of the migratory potential of limb muscle progenitor cells, but further studies are necessary.

Our previous research revealed the presence of Pax7-positive cells in myotomal muscles during early stages (stage 24) of sand lizard development (Rupik et al. 2012). It is noteworthy that we did not observe Pax7-positive cells in analyzed stages of limb bud development. Similar results were obtained by Lee et al. (2013) in the early rat forelimb. Pax7 expression was not observed in the limb muscle mass, whereas this protein was clearly present in the dorsal neural tube and the dermomyotome. During later developmental stages, Pax7 protein was detected in the central and basal part of the forelimb bud, then Pax7-positive cells were observed in the dorsal and ventral limb bud mass. The spatiotemporal expression of Pax3 and Pax7 proteins was also noted in the mouse limb, in which Pax3 expression precedes the expression of Pax7 (Relaix et al. 2004). These data strongly confirm the hypothesis that Pax3 protein plays a crucial role in primary myogenesis, whereas Pax7 is required later for the maintenance of satellite cells (Tajbakhsh et al. 1997; Seale et al. 2000). Based on our results, we suggest that the Pax3/Pax7 pattern expression during limb bud muscle development is conserved in amniotes.

### Expression of MyoD and Myf-5 proteins during early limb bud development

It is commonly known that Myf5 and MyoD are involved in the shift from proliferating myoblasts to multinucleated muscle fibers capable of contractions. It has been suggested that the activation of MyoD and Myf5 is controlled in developing myoblasts by their degradation (Thayer et al. 1989), modification (Lindon et al. 1998), signaling (Vaidya et al. 1989; Li et al. 1992), or interference with heterodimerization (Benezra et al. 1990).

Western blot analysis of sand lizard limb buds revealed the presence of MyoD and Myf5 proteins in all investigated developmental stages (stages 22–32). It has been demonstrated that in chicks and mice during early developmental stages of limb buds Myf5 is the first MRF protein expressed. As myogenesis proceeds, Myf5 was no longer detectable, but MyoD was observed. In contrast to chicks and mice, during rat limb bud development, MyoD is the first MRF protein detectable (Lee et al. 2013; Mok et al. 2015). Although our studies revealed the presence of MyoD and Myf5 proteins in all analyzed stages, we cannot exclude spatiotemporal pattern expression of the mentioned proteins because of asynchronous myogenesis in sand lizard limb buds, i.e., in the limb bud myogenic pool the muscle cells are at different stages of differentiation: mitotically active premyoblasts, myoblasts, and myotubes were observed.

It is commonly accepted that MyoD induces the myogenic program only as homo- or heterodimers with other helix–loop–helix transcription factors such as E12 or E47 (Maleki et al. 1997). Sand lizard limb bud Western blot analysis revealed the presence of two bands of MyoD protein corresponding to monomeric (mMyoD) and dimeric (dMyoD) fractions. Statistical analysis showed significant differences between the levels of mMyoD and dMyoD at a later developmental stage (stage 32). A higher level of mMyoD suggests a decrease in the number of undifferentiated cells in the limb bud myogenic pool.

It has been reported that Myf5 is a “determination” factor expressed in proliferating myoblasts (Braun et al. 1989). Lindon et al. (1998) found that Myf5 is downregulated in cells undergoing differentiation. The authors suggested that the Myf5 level in proliferative cells is strongly regulated by cell cycle–associated events that include the degradation of this factor during mitosis. Several studies revealed that the cell cycle depends on the Myf5 level and its phosphorylation (Lindon et al. 1998; Song et al. 1998; Kitzmann et al. 1998; 1999; Tintignac et al. 2000). Previous data suggested that Myf5 undergoes phosphorylation by a mitosis-specific kinase(s), and that this modified Myf5 is highly unstable in mitotic cells and is rapidly degraded, probably by a 26-S proteasome-dependent mechanism (Lindon et al. 1998). Based on the results from Ohya et al. (2006), *Myf5* in turtles undergoes alternative splicing (twelve nucleotides deletion comparing it with the sequences of other vertebrate *Myf5* genes). However, the authors suggest that the mentioned deletion is only turtle lineage–specific feature. The sand lizard limb bud western blot analysis revealed two separate bands (~ 30 kDa) of Myf5 protein. We assume that the observed bands correspond to non-phosphorylated (Myf5) and phosphorylated (pMyf5) fractions of Myf5. The conducted analysis showed statistically significant differences between Myf5 and pMyf5 at stages 24 and 28. Therefore, the limb bud myogenesis is asynchronous; both non-phosphorylated and phosphorylated fractions of Myf5 could be observed. However, it is not excluded that separated bands were represented proteins translated on two Myf5 isoforms, future studies will clarify this.

It is now clearly established that limb myogenesis shares many similar features among all vertebrates. During limb myogenesis, mononucleated myoblasts elongate and differentiate into mononucleated myotubes accompanied by mononucleated cells (premyoblasts or myoblasts). Similar to other tetrapods, the VLLs of the dermomyotome are a source of limb muscle progenitor cells. Our studies on the sand lizard for the first time revealed the presence of Lbx2 protein in progenitor muscle cells migrating to the limb bud. Despite the fact that in all vertebrates genetic control of muscle fiber differentiation includes the same transcription factors (Pax3/7, MyoD, Myf5), the known differences in myogenic genetic control, observed in mouse, chick, rat, and sand lizard, only concern their spatiotemporal expression.

## Electronic supplementary material

Fig. S1 LC-MS based identification of Lbx peptides from *Lacerta agilis* limb buds extract. **A.** The limb bud lysates from *Lacerta agilis* embryos (Stages 21 and 24) were separated by SDS-PAGE and stained by modified Silver Staining (Shevchenko et al. 1996). Stained portion of the gel were cut out (black box). The size of cutout bands corresponded to the size of bands detected in Western blot technique (see Fig. 5A). Samples of proteins were sent for the identification by an LC-MS method in the Mass Spectrometry Laboratory, IBB PAS and searched with MASCOT (Matrix Science). **B.** Protein sequence alignment of *Xenopus laevis* Lbx1 (Lbx1; accession number NP_001089192.1), epitope (Ep) recognized by commercially available antibody (mouse monoclonal anti-Lbx2; Abcam), identified peptide 1 (P1), identified peptide 2 (P2), and identified peptide 3 (P3). The comparison identified peptides with the NCBI protein sequences database was carried out using MASCOT program. Obtained results revealed the presence of three peptides corresponding to regions present in *X. laevis* Lbx1 amin acid sequence. Sequence alignment: ClustalW2, http://www.ebi.ac.uk (PNG 1377 kb)

High resolution image (TIF 9848 kb)
