# Supplementary figures and images for: Primary myogenesis in the sand lizard (Lacerta agilis) limb bud
Source: Dev Genes Evol. 2019 Jun 18;229(5):147–59. doi: 10.1007/s00427-019-00635-7 (PMC6867991; doi:10.1007/s00427-019-00635-7)

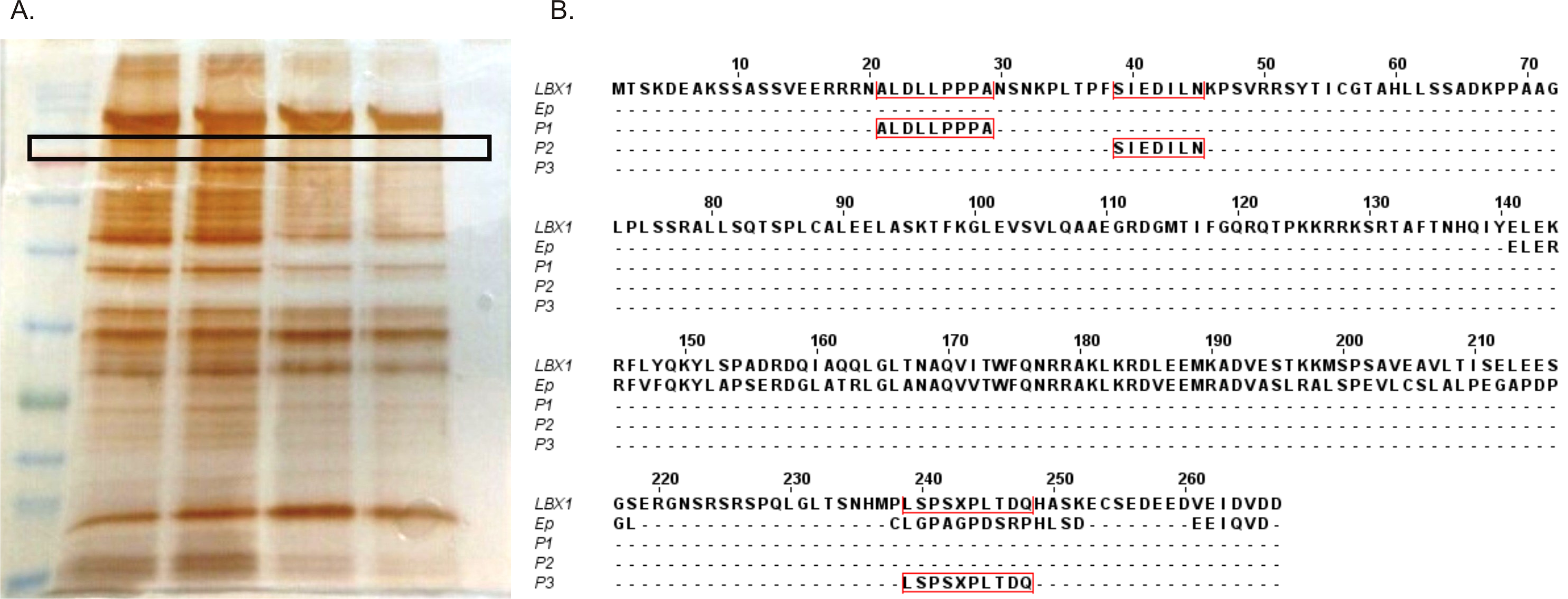

Supplement: Supplementary file 1 — LC-MS based identification of Lbx peptides from Lacerta agilis limb buds extract. A. The limb bud lysates from Lacerta agilis embryos (Stages 21 and 24) were separated by SDS-PAGE and stained by modified Silver Staining (Shevchenko et al. 1996). Stained portion of the gel were cut out (black box). The size of cutout bands corresponded to the size of bands detected in Western blot technique (see Fig. 5A). Samples of proteins were sent for the identification by an LC-MS method in the Mass Spectrometry Laboratory, IBB PAS and searched with MASCOT (Matrix Science). B. Protein sequence alignment of Xenopus laevis Lbx1 (Lbx1; accession number NP_001089192.1), epitope (Ep) recognized by commercially available antibody (mouse monoclonal anti-Lbx2; Abcam), identified peptide 1 (P1), identified peptide 2 (P2), and identified peptide 3 (P3). The comparison identified peptides with the NCBI protein sequences database was carried out using MASCOT program. Obtained results revealed the presence of three peptides corresponding to regions present in X. laevis Lbx1 amin acid sequence. Sequence alignment: ClustalW2, http://www.ebi.ac.uk (PNG 1377 kb) [file 427_2019_635_Fig7_ESM.png]

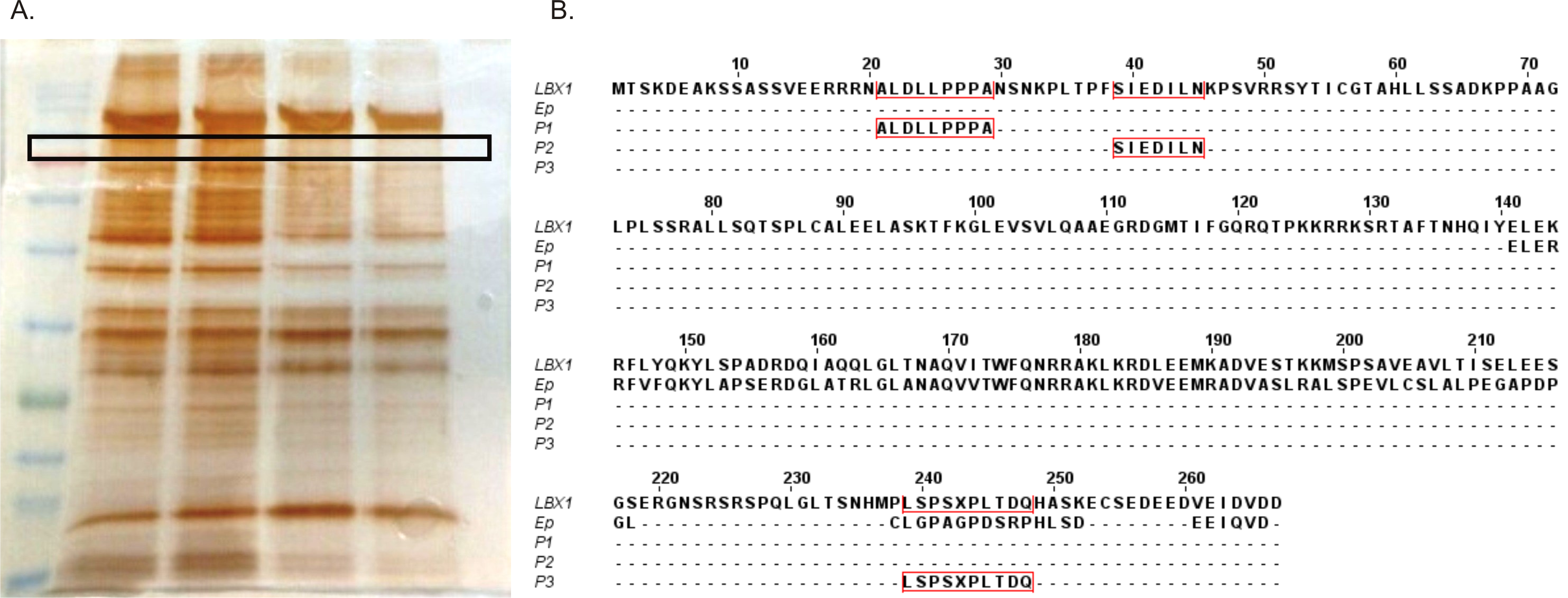

Supplement: Supplementary file 2 — High resolution image (TIF 9848 kb) [file 427_2019_635_MOESM1_ESM.tif]
